# The state of Ghanaian liver medicine

**DOI:** 10.11604/pamj.2021.39.148.20271

**Published:** 2021-06-24

**Authors:** Adwoa Agyei-Nkansah, Simon Taylor-Robinson

**Affiliations:** 1 Department of Medicine, School of Medicine and Dentistry, University of Ghana, Accra, Ghana,; 2 Department of Surgery and Cancer, Imperial College London, St. Mary’s Hospital Campus, London W2 1NY, United Kingdom

**Keywords:** Liver, challenges, management, Ghana

## Abstract

This paper aims to highlight the challenges in managing liver diseases in Ghana and the efforts needed to improve services to help curb the high rate of liver mortality in the young adults. Ghana is a rising, middle-income West African country with well-established administrative systems for healthcare, albeit with hospitals lacking modern equipment and being devoid of infrastructure for sophisticated interventional procedures. Although liver disease is common, due to the high prevalence of chronic viral hepatitis B and C infection, antiviral drugs are commonly unavailable, even in the rare instances where they can be afforded. Hospital wards and outpatient clinics are usually over-crowded with long waiting times and limited doctor-patient time interaction. Treatment for end-stage liver disease can be a challenge, with limited endoscopic services, which are centered in the big cities and with expertise which is not widespread. The training program in endoscopic therapies by the Mayo Clinic (Rochester, Minnesota, USA), with faculty coming to Ghana to disseminate practical skills during a “training of trainers” program has gone a long way spreading the knowledge of managing life-threatening complications, such as variceal hemorrhage, albeit on a small scale in national terms. Collaboration between institutions from well-resourced and poorly-resourced countries exemplifies how such partnerships can go a long way in helping to support local training needs and the development of transferrable skills. Such partnerships may effectively provide healthcare workers with adequate training, with hepatology treatment protocols that are adapted to the local environment and thus allowing contextualisation of generic guidelines from the developed world and making them applicable to local settings.

## Essay

Ghana is a low middle-income country in West Africa and in the colonial era, it was known as the Gold Coast, a name that harked back to the great Asante kingdom, based around the Central Ghanaian city of Kumasi. The nascent nation regained its independence from its British colonial masters in 1957. Ghana´s population stands at about 27 million people. Despite its relatively poor economic state, it has a rich culture and history. In West Africa, Ghanaians are known for having the highest literacy rates in the region and a good educational system. The country, with its plentiful natural resources, such as cocoa, gold, bauxite, timber and oil, has a higher gross domestic product than most of its West African neighbours, but still, it depends heavily on donor funding from international financial institutions, with technical aid from both foreign governmental and non-governmental sectors.

Unusually for sub-Saharan Africa, Ghana has taken significant steps in the past two decades towards true democracy under a multiparty system, the post-colonial period having been peppered with several military coups. The country abuts the Gulf of Guinea and Atlantic Ocean and is 4° north of the Equator, encompassing about 238,537 km^2^ of land [1]. In 2019, Ghana's population was approximately 31.1 million. About 56.1% of the population is urban with a life expectancy of 63.8 and 66.1 years for males and females respectively [2]. The World Bank income classification declared Ghana as a lower middle income country (LMIC) in 2013 with about 24.2% of the population living below the poverty line [3]. The total expenditure on health per percentage of gross domestic product (GDP) was 3.6% as of 2014 [3]. Of note, the country is mainly agrarian, with about 59% of the population being farmers [3].

Ghana´s doctor-to-patient ratio stands at only one doctor to 10,452 patients in 2012. In 2000, the World Health Organization (WHO) ranked Ghana´s overall health system performance as 135^th^ out of the 191 WHO member states. Despite this, the country has a relatively high literacy rate for a developing country. Given that it is well-endowed with natural resources, compared to most West African countries, Ghana´s per capita economic output is higher than almost all its sub-Saharan counterparts. This may act as a driver for change in the healthcare system. With political stability over the past two decades, it is time for Ghana to take major strides toward accessible healthcare for all.

**The structure of the Ghanaian health sector:** the Ghana health sector is overseen by two governmental agencies. The Ministry of Health (MOH) and the Ghana Health Services (GHS). Policy formulation, monitoring and evaluation of health service delivery and the development of the framework for food and drugs regulation in the health service are all under the umbrella of the MOH, based in Accra, the capital city. The GHS, on the other hand, is responsible for service provision of the healthcare system across the country. The GHS mandate includes implementation of national healthcare policies, provision and management of health care resources. Healthcare delivery in Ghana comprises of primary outreach in both rural and urban settings, secondary facilities with clinics offering basic treatment and tertiary healthcare institutions, usually in the form of regional and university-affiliated hospitals in the larger cities, such as Accra, Kumasi, Tamale and Cape Coast. Such tertiary care provides specialist treatment and is mainly limited to specific hospitals in the big cities.

**Burden of liver disease:** chronic liver disease is known to occur throughout the world, cirrhosis being defined histologically as fibrosis and architectural distortion of the liver parenchyma with formation of regenerative nodules. This usually results from a variety of underlying chronic liver diseases, ranging from viral hepatitis and the effects of long-term excess alcohol consumption, through to autoimmune liver disease and the hepatic manifestations of the metabolic syndrome. Cirrhosis is the ultimate stage for all these liver conditions with all-cause mortality from liver disease currently being ranked fifth as a cause of death in many countries around the world. Epidemiological studies have shown that chronic viral hepatitis B (CHB) is hyper endemic in sub-Saharan Africa and some parts of Asia with a prevalence rate ranging from 5-10% of the adult population and this is a particular problem in West African countries, such as Ghana [4]. Strikingly, 40% of patients with cirrhosis in Ghana were found to have hepatitis B in a study conducted by Blankson *et al*. [5]. In Ghana viral hepatitis is known to be more prevalent than human immunodeficiency virus (HIV), leading to significant morbidity and mortality in terms of potentially avoidable liver disease. This is partly due to late presentation by the patient population, owing to lack of recognition and insufficient public health education. Given the good general education of the population, it is a regrettable fact that chronic hepatitis B infection remains in the hyper endemic category in Ghana with a prevalence of greater than 8%. Alarmingly, the prevalence of hepatitis B using hepatitis B surface antigen (HBsAg) screening in a study conducted by Ofori-Asenso and colleagues was as high as 12.3% in the general Ghanaian population [6]. This reinforces the need for public health campaigns across the country, with the raising of general awareness of hepatitis B as a potentially preventable problem through uptake of the birth-dose hepatitis B vaccine and improved public health measures from safe-sex campaigns, maternal hepatitis B screening through to effective blood transfusion screening and proper sterilization of legitimately reusable hospital equipment.

Chronic infection with hepatitis B increases the individual risk of developing liver complications such as cirrhosis and liver cancer. Up to a third of chronically infected patients are estimated to develop chronic liver inflammation (hepatitis), out of which, up to half may develop cirrhosis. In the Ghanaian context, primary liver cancer (hepatocellular carcinoma - HCC) is a condition with dire consequences and with few treatment modalities. There is limited access to regular HCC screening using ultrasound in the chronic liver disease population and for those who do succumb, options for effective palliative care are lacking, while highly prevalent comorbidities, such as HIV/AIDS and risk factors, such as aflatoxin exposure and alcohol misuse all play a role in explaining why liver cancer has a much higher morbidity rate than in developed countries. Yang and colleagues found in their study that HCC due to chronic viral hepatitis B infection occurred earlier in sub-Saharan Africans with an average age range of 32.5-37.5 years, compared to 57.5-62.5 years for hepatitis C-associated liver cancer. Cumulatively, with respect to hepatitis B virus (HBV) carriers, 2% developed liver cancer before age 20 years, 13% by age 30 years and 43% before 40 years of age. The estimated prevalence from some studies gave a prevalence of hepatitis C infection between 2.4% to 3.0% [7]. In Accra, the capital city, cirrhosis was found to be the leading cause of liver related mortality. An autopsy audit on liver diseases in Ghana observed that at autopsy, cirrhosis was commonly found and listed as a cause of death [8]. Cirrhosis has been difficult to estimate in Ghana owing to several factors. Contributing factors include insufficient capably trained doctors who can perform liver biopsy, patient reluctance to undergo the invasive procedure and lack of sufficiently-equipped diagnostic histopathology facilities across the country to interpret results if available.

A key challenge in the clinical care of liver cancer is the lack of safe, reliable and cost-effective means of diagnosing and monitoring treatment response. Early diagnosis is important for improving prognosis by identifying disease early enough to permit timely treatment. Currently, the imaging tools used to detect disease is ultrasonography (US, computerized tomography (CT) and magnetic resonance imaging (MRI)). Although relatively reliable, these methods require advance scheduling, are costly and not available across the country, except for in a few tertiary centres. Detecting liver cancer at an early stage, as well as identifying recurrence early to allow for timely retreatment is key to a better prognosis and prolonged survival. Standard grey-scale ultrasound can simply provide subjective visual information on liver tissue appearance, rather than providing objective information on liver fibrosis, through measurement of liver stiffness (unlike techniques such as transient elastography including Fibroscan™). Standard ultrasound can only detect the presence of cirrhosis at a relatively late stage when liver tissue morphology is more obvious to the eye of the radiologist. Non-invasive, safe, low-cost and highly reliable imaging techniques have been developed, such as ultrasound-based transient elastography, using the FibroScan™ machine (Echosens, Paris, France). This technology provides measurements of liver stiffness as a proxy for the severity of fibrosis, in addition to information on hepatic fat content through the measurement of the controlled attenuation parameter (CAP) score. This holds tremendous promise in the clinical care of these patients around the world, but FibroScan™ is currently non-existent in Ghana.

**Treatment of liver disease:** most developing nations with constrained resources have to deal with numerous health issues. The constrained resources and basic health infrastructure result in most patients in these countries having little or no screening, diagnostic or treatment opportunities for their liver disease and management of its complications. Most of these patients presents with late disease that is not amenable to cure. Several factors account for the delay in presentation, but commonly these include financial challenges, a lack of education and insight into the disease, religious beliefs and the distance to travel to get to the nearest health facility, leading to people seeking care from the traditionalists instead. Despite their high medical, psychosocial and economic impact, chronic viral hepatitis which together accounts for 80-90% of all HCC worldwide, these conditions have not received the needed attention in Ghana. Currently, the cost of treating HBV in Ghana using the generic oral antivirals is about GHC 200-400 (USD 30-80) a month. The prohibitive cost of these antivirals has made supplements with little proven benefit, like silymarin, livolin forte, liv52 and Chinese herbal remedies very popular in the country. In the context of HIV, HIV/HBV co-infected patients are eligible for free treatment, making it more advantageous to be co-infected from both a medical and financial point of view. It is of note that HIV with a nation-wide prevalence in Ghana of 2.4% in 2016 constituted a Millennium Development Goal challenge, bringing in millions of dollars from funding agencies and Ghana development partners, while in stark contrast, chronic hepatitis B with a prevalence of 12.3% has been largely ignored.

Direct acting antivirals for hepatitis C treatment are known to produce up to 99% cure rate, but the cost of treating this condition is outside the reach of the average Ghanaian, costing between 800 to 1,200 USD for a 3-month course of the generic form of the available drugs. The cost of treatment of these conditions is outside the pockets of most Ghanaians. Publicly funding this will have a huge economic impact on health expenditure. In 2003, Ghana introduced the national health insurance scheme, with the main objective of eliminating out-of-pocket payments and improving healthcare access, but screening, diagnosis, treatment and vaccination against hepatitis B outside the expanded program on immunization are not covered under the scheme. One of the effective ways of preventing mother-to-child transmission of hepatitis B is timely hepatitis B birth-dose vaccination. This should be performed at the time of birth to prevent any viral transmission. Currently, new-born infants in Ghana receive hepatitis B vaccination as part of the pentavalent regime at 6, 10 and 14 weeks after birth, but the birth dose is lacking. This implies that babies born to hepatitis B positive mothers are highly at risk of the infection from day one of birth until 6 weeks, by which time most infants will carry the virus. It therefore presents a missing opportunity in Ghana´s response to the burden of the disease, since mother-to-child transmission is the most common mode of transmission in the West African context. The prevalence and transmission routes of hepatitis B infection makes it a public health problem of prime importance in Ghana. This calls for urgent public health intervention and policy directions for curbing the hepatitis B problem, to reduce its psychosocial and economic burdens on Ghanaians and to avert any potential future explosion in prevalence.

The rising incidence of obesity and diabetes throughout the world will account for more liver cancer in the future. A scoping of the literature identified several population-based studies that recorded a significantly increased incidence of liver cancer in obese and diabetic patients. There is also increasing evidence that suggests an accelerated risk of liver cancer in non-alcoholic fatty liver disease (NAFLD) patients. Under these circumstances, NAFLD-related liver cancer incidence is expected to go up in the future. There is no wide-scale screening for HCC on-going in the risk group due to inadequate resources in Ghana; consequently, most patients present with late disease, leading to a high mortality almost approximating to incidence. The other challenge in helping control cancer in developing countries is that treatment options are very limited and even if cases are detected early, there is usually very little that one can offer; even in cases where treatments are available, they are expensive in the Ghanaian context.

The contribution of the pharmaceutical companies in facilitating affordable access to treatment especially in developing countries cannot be over-emphasized. Most research conducted by pharmaceutical companies is in the developed countries where participants often enjoy free treatment in areas where they have a comprehensive insurance cover. Lots of advocacy should be done to lobby the pharmaceutical companies to increase the number of open-access programs of new treatments to developing countries. The now annual training programme in endoscopic therapies by the Mayo Clinic (Rochester, Minnesota, USA) with faculty coming to Ghana to impact skills during the training of trainers has gone a long way in managing complications, such as variceal haemorrhage, albeit on a small scale in national terms. Clinicians require training in the use of these new diagnostic tools and in making informed choices for treatment initiation and monitoring and in adequate and timely clinical decision making. Moreover, there is a need to contextualize generic guidelines making them applicable to local settings. Twinning programs offer interdisciplinary and interactive training on clinical aspects of drug resistant Tuberculosis diagnosis (DR TB) diagnosis and care. It consists of an eight-week online training (3-4 hours/week) followed by two weeks face-to-face.

**Future developments:** developing and developed countries have significantly varying incidence and mortality of liver diseases. The quantum in developing countries cannot be accurately estimated due to scanty and unreliable statistics. This has led to assumptions being made as to the prevalence of the disease burden, which can be misleading. Equipping hospitals, staff training, public education and development of appropriate and locally acceptable protocols in addition to cheaper forms of care are vital, as is the need to formulate policies at both local and international level. There is always the dying need for a low-cost intervention that could potentially save many lives in the future. International advocacy is needed to spur the international community and individual governments to lead the change. Lobbying of governments and Health Ministry should be effected to give a clear picture of the effect of ignoring these diseases, especially liver cancer and the impact it will have on the health infrastructure and the economically-productive age group in the near future.

**Capacity building and strengthening health workforce:** one major challenge in providing adequate health care is the dwindling nature of trained healthcare professionals. With the aging population, there is increased demand on healthcare coverage, but this is limited by the low numbers of health professionals and the en masse retiring of some specialty and nursing staff, owing to a blip in the demographics as the people of the baby-boomer generation get to the end of their working lives. There should be regular on-the-job training of health care workers. Having genuine long-term, equitable twinning partnerships between well-resourced and under resourced countries institutions will be beneficial in providing support in training and education as well as skills transfer for the developing country. These involve sharing of knowledge and expertise with the ultimate aim of improving healthcare delivery in resource-constrained countries. These projects should usually be led by local experts and fashioned in such a way to fit local resources and needs.

The ELMA foundation, a Clinton health access initiative planning grant is a 7-year partnership with the government of Rwanda, focusing on transferring knowledge, the formation of a residency health management program and the adoption of a raft of other programs. This involved each school sending delegates annually to work with local faculty in teaching and service delivery [9].

The M-PACT West Africa College of Physicians - Royal College of Physicians of London joint programme is another example of such partnership and this has helped to improve management of a variety of diseases including HIV, malaria and tuberculosis [10]. Health partnerships through the Tropical Health Education Trust (THET), collaboration with industry and Pharma on Fibroscan™ provision and on affordable treatments, such as tenofovir for hepatitis B, will help bridge the treatment gap. This is more important, given the World Health Assembly goal for elimination of viral hepatitis by 2030.
